# Supplementary figures and images for: 5th generation vs 4th generation troponin T in predicting major adverse cardiovascular events and all-cause mortality in patients hospitalized for non-cardiac indications: A cohort study
Source: PLoS One. 2021 Feb 9;16(2):e0246332. doi: 10.1371/journal.pone.0246332 (PMC7872231; doi:10.1371/journal.pone.0246332)

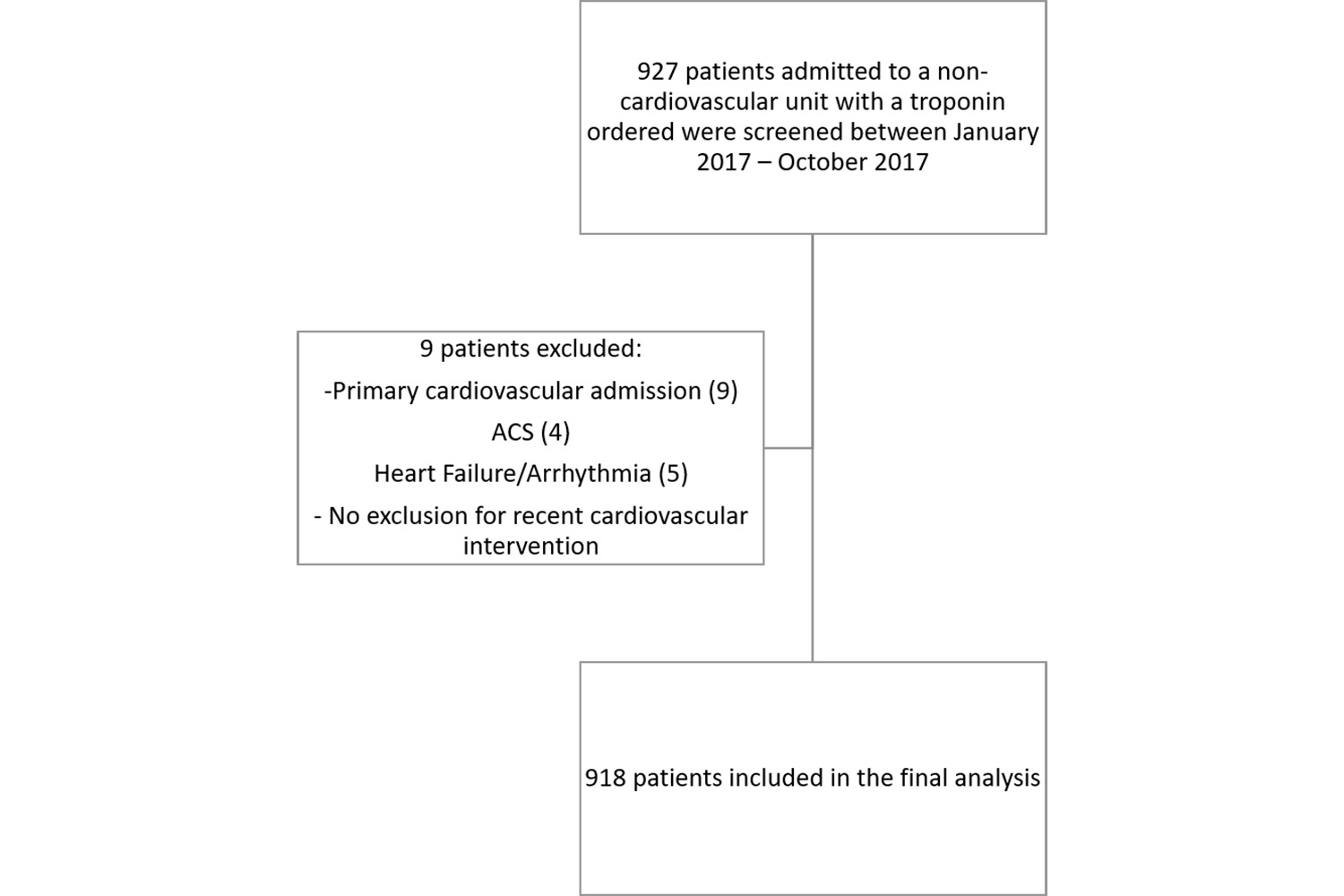

Supplement: S1 Fig — (TIF) [file pone.0246332.s001.tif]

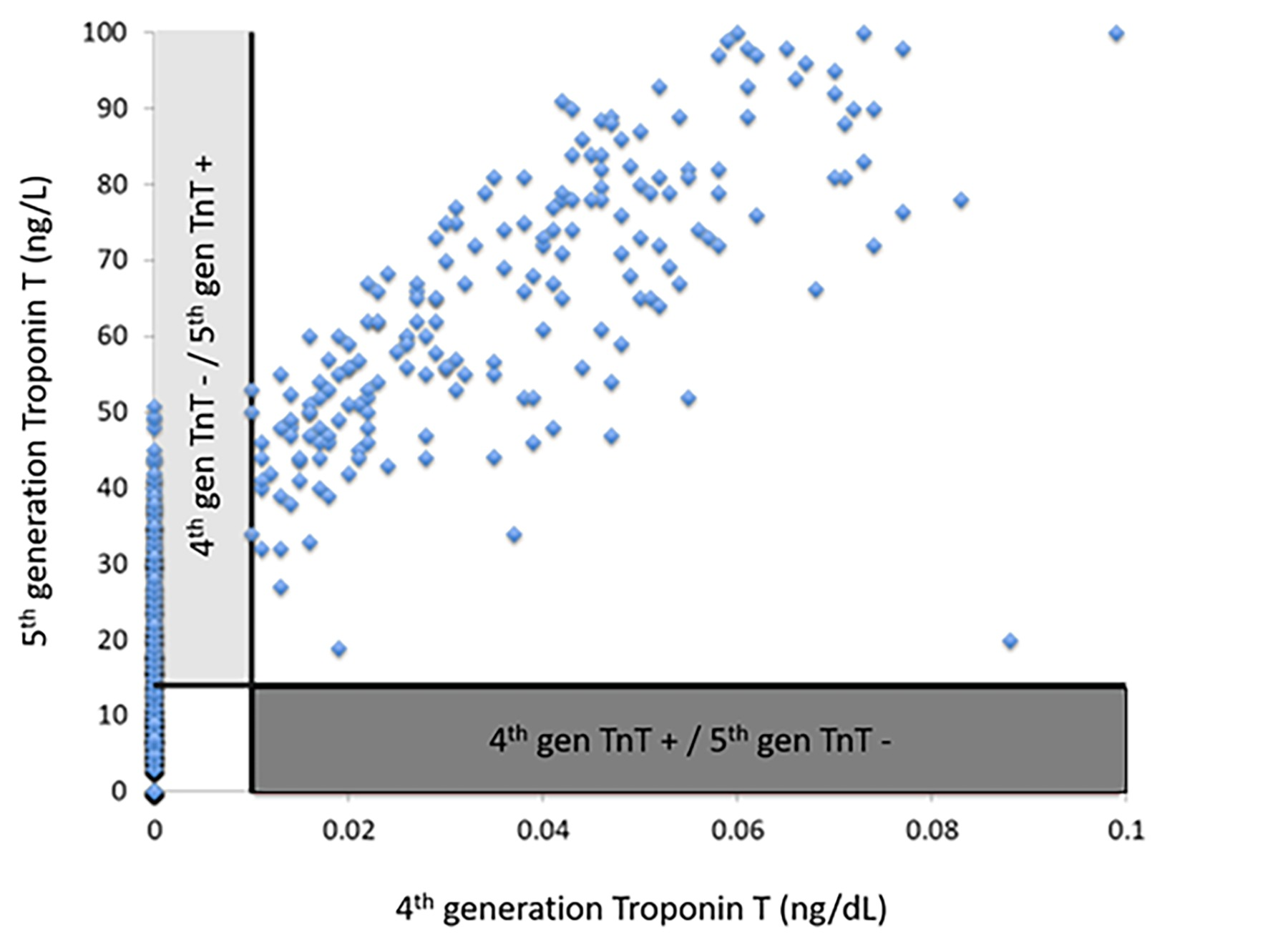

Supplement: S2 Fig — Per sample correlation between 4th and 5th generation Troponin T assays. No patients had a sample that was positive on the 4th generation Troponin T assay but was negative on the 5th generation troponin T assay. (TIF) [file pone.0246332.s002.tif]

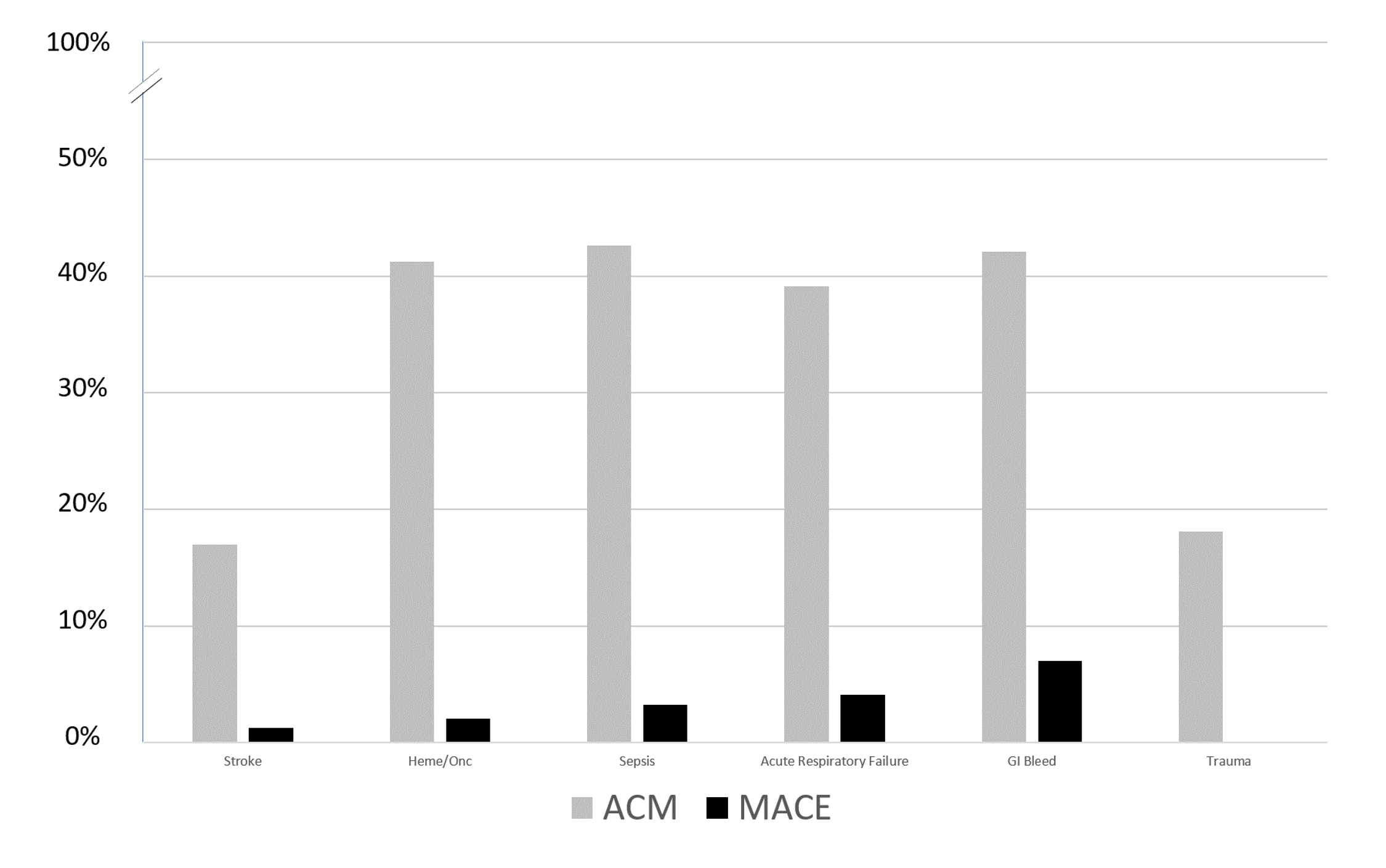

Supplement: S3 Fig — ACM and MACE rates by reason for admission. No statistical analysis was run due to low event rates. (TIF) [file pone.0246332.s003.tif]
