# Supplementary material for: 5th generation vs 4th generation troponin T in predicting major adverse cardiovascular events and all-cause mortality in patients hospitalized for non-cardiac indications: A cohort study
Source: PLoS One. 2021 Feb 9;16(2):e0246332. doi: 10.1371/journal.pone.0246332 (PMC7872231; doi:10.1371/journal.pone.0246332)
Supplement: S2 Table — (DOCX) [file pone.0246332.s005.docx]

**S2 Table. Sensitivity, specificity, positive predictive value, negative predictive value and accuracy of 4^th^ and 5^th^ generation troponin T in predicting MACE.**

|  | **Sensitivity** | **Specificity** | **PPV** | **NPV** | **Accuracy** |
| --- | --- | --- | --- | --- | --- |
| **4^th^ gen TnT** | 77% | 59% | 6% | 99% | 59% |
| **5^th^ gen TnT** | 90% | 33% | 4% | 99% | 35% |
