# Supplementary material for: 5th generation vs 4th generation troponin T in predicting major adverse cardiovascular events and all-cause mortality in patients hospitalized for non-cardiac indications: A cohort study
Source: PLoS One. 2021 Feb 9;16(2):e0246332. doi: 10.1371/journal.pone.0246332 (PMC7872231; doi:10.1371/journal.pone.0246332)
Supplement: Attachment — Submitted filename: Response to Revisions PLOS.docx [file pone.0246332.s007.docx]

**Response to Reviewers**

Editor Comments

1. Please ensure that your manuscript meets PLOS ONE's style requirements, including those for file naming. The PLOS ONE style templates can be found at

https://journals.plos.org/plosone/s/file?id=wjVg/PLOSOne_formatting_sample_main_body.pdf and

https://journals.plos.org/plosone/s/file?id=ba62/PLOSOne_formatting_sample_title_authors_affiliations.pdf

**Thank you for providing these, and our apologies for not doing this initially. We have modified the Title page and text to conform to these guidelines.**

2. In your Methods section, please provide additional information about the participant recruitment method and the demographic details of your participants. Please ensure you have provided sufficient details to replicate the analyses such as descriptions of where participants were recruited and where the research took place (location/hospital/site).

**Thank you for your recommendation. We have added some details in the first paragraph of the Methods section (under Study Population) specifically to allow for more reproducibility.**

3. Please provide a sample size and power calculation in the Methods, or discuss the reasons for not performing one before study initiation.

**Given the study design, where each participant served as their own control and was included in both the study arm (5^th^ generation TnT) and the control arm (4^th^ generation TnT), there was not a traditional sample size calculation undertaken as the event rate would be the same in both arms. However, based rates of all-cause mortality and major adverse cardiovascular event rates on previous studies done at our institution using a similar definition of outcomes, we predicted 1000 patients would be needed to show adequate events. This has been added under the Methods section (under study population).**

4. We note that you have indicated that data from this study are available upon request. PLOS only allows data to be available upon request if there are legal or ethical restrictions on sharing data publicly. For information on unacceptable data access restrictions, please see http://journals.plos.org/plosone/s/data-availability#loc-unacceptable-data-access-restrictions.

In your revised cover letter, please address the following prompts:

a) If there are ethical or legal restrictions on sharing a de-identified data set, please explain them in detail (e.g., data contain potentially identifying or sensitive patient information) and who has imposed them (e.g., an ethics committee). Please also provide contact information for a data access committee, ethics committee, or other institutional body to which data requests may be sent.

b) If there are no restrictions, please upload the minimal anonymized data set necessary to replicate your study findings as either Supporting Information files or to a stable, public repository and provide us with the relevant URLs, DOIs, or accession numbers. Please see http://www.bmj.com/content/340/bmj.c181.long for guidelines on how to de-identify and prepare clinical data for publication. For a list of acceptable repositories, please see http://journals.plos.org/plosone/s/data-availability#loc-recommended-repositories.

**There are no restrictions, and we have uploaded a data set.**

Reviewer Comments

Reviewer #1: This is a well-written, well-organized and well-illustrated paper. It presents the results of original research and makes a valuable contribution to knowledge and understanding of cardiac biomarkers. In particular, authors compare 5th generation versus 4th generation troponin T in predicting major adverse cardiovascular events and all-cause mortality in patients hospitalized for non-cardiac indications. They found that 5th generation TnT is more sensitive and less specific in predicting both all-cause mortality and major adverse cardiovascular.
I have just a few small comments on the text, which the authors may wish to address:
• I recommend avoiding the use of the abbreviation the first time it was used in the text. After you define an abbreviation (regardless of whether it is in parentheses), use only the abbreviation. Do not alternate between spelling out the term and abbreviating it.

**Thank you for this note. We have reviewed and minimized the use of abbreviations, and only used the abbreviations once defined.**
• I suggest to text-align

**We have changed the formatting to justified text align.**
• According to submission guidelines, do not use non-standard abbreviations unless they appear at least three times in the text.

**Thank you for pointing this out, and we agree. We have decreased the use of abbreviations only to those terms used repeatedly to simply.**
• I suggest to remove the comma before to cite reference in the text.

**Thank you. We have reviewed the comma before in-text citations.**
• References should be formatted as indicated by the guidelines (i.e. Hou WR, Hou YL, Wu GF, Song Y, Su XL, Sun B, et al. cDNA, genomic sequence cloning and overexpression of ribosomal protein gene L9 (rpL9) of the giant panda (Ailuropoda melanoleuca). Genet Mol Res. 2011;10: 1576-1588).

**We have reviewed the formatting of the references for consistency.**
Finally, a note on the completeness of the bibliography; in order to improve the bibliography I suggest completing the list of references with the following excellent articles: PMID: 28262193, PMID: 21288172, PMID: 19732763.

**Thank you for directing us to these articles. They have been added.**
I recommend publication of this work immediately in PlosOne following consideration of the minor points above.

Reviewer #2: The manuscript is very interesting and well-written. However, I have some minor suggestions to improve its quality.
In the introduction, in the first sentence I suggest to replace preferred with recommended by guidelines.

**We agree and have changed the verbiage in paragraph 1 of the introduction.**
The main limitation is that the paper does not report seminal studies in this field. Please, consider citing the following: PMID: 29698621, PMID: 31223265, PMID: 29180917

**Thank you for directing us to these articles. They have been added.**
